# Cutaneous manifestations of bartonellosis^☆^^☆☆^

**DOI:** 10.1016/j.abd.2019.09.024

**Published:** 2019-10-02

**Authors:** Karina de Almeida Lins, Marina Rovani Drummond, Paulo Eduardo Neves Ferreira Velho

**Affiliations:** a Department of Clinical Medicine, School of Medical Sciences, Universidade Estadual de Campinas, Campinas, SP, Brazil; b Laboratory of Applied Research in Dermatology and Bartonella Infection, School of Medical Sciences, Universidade Estadual de Campinas, Campinas, SP, Brazil; c Discipline of Dermatology, Department of Clinical Medicine, School of Medical Sciences, Universidade Estadual de Campinas, Campinas, SP, Brazil

**Keywords:** *Bartonella*, Skin diseases, Neglected diseases

## Abstract

Bartonellosis are diseases caused by any kind of *Bartonella* species. The infection manifests as asymptomatic bacteremia to potentially fatal disorders. Many species are pathogenic to humans, but three are responsible for most clinical symptoms: *Bartonella bacilliformis*, *Bartonella quintana*, and *Bartonella henselae*. Peruvian wart, caused by *B. bacilliformis*, may be indistinguishable from bacillary angiomatosis caused by the other two species. Other cutaneous manifestations include maculo-papular rash in trench fever, papules or nodules in cat scratch disease, and vasculitis (often associated with endocarditis). In addition, febrile morbilliform rash, purpura, urticaria, erythema nodosum, erythema multiforme, erythema marginatus, granuloma annularis, leukocytoclastic vasculitis, granulomatous reactions, and angioproliferative reactions may occur. Considering the broad spectrum of infection and the potential complications associated with *Bartonella* spp., the infection should be considered by physicians more frequently among the differential diagnoses of idiopathic conditions. Health professionals and researchers often neglected this diseases.

## Introduction

Bartonellosis are diseases caused by any *Bartonella* species.^1^ They are neglected, re-emergent, and distributed worldwide, affecting mainly populations suffering from poverty, with precarious sanitation, and that are in direct contact with arthropods and domestic animals.^2^, ^3^ Most species cause zoonotic diseases.^1^, ^3^

*Bartonella* spp. are fastidious Gram-negative bacilli, well adapted to a variety of animal reservoirs, particularly mammals. These bacteria are capable of infecting and surviving inside erythrocytes. The intraerythrocytic phase allows for a protection niche for the agent, resulting in a prolonged and recurrent infection.^4^ The bacteria can also infect endothelial cells.^5^

The main route of transmission of *Bartonella* spp. is from infected humans or animals to new hosts through blood-sucking arthropod vectors. Transmission through animal scratches has been reported but it is not certain, since fleas are needed for transmission among cats.^1^, ^6^ Recent studies reinforce the hypothesis that these bacteria can be transmitted through blood transfusion, which is a concern for people all over the world since currently there is no preventive action against this possibility.^3^, ^7^, ^8^, ^9^ In addition, asymptomatic infection by *Bartonella* sp. has already been detected in blood donors.^3^, ^8^, ^9^, ^10^, ^11^, ^12^, ^13^, ^14^, ^15^, ^16^, ^17^, ^18^

*Bartonella* spp. are responsible for a broad clinical spectrum, from asymptomatic bacteremia to potentially fatal presentations. Although the manifestations associated with bartonellosis have increased considerably over the past decades, physicians usually do not consider the possibility of infection with these bacteria among differential diagnoses, except in cases with localized lymph node enlargement or endocarditis with negative culture,^19^, ^20^ which suggests that bartonellosis has been neglected by the medical community, leaving many cases undiagnosed.

## Clinical aspects

Among the 16 species of *Bartonella* that are pathogenic to humans, three are responsible for the majority of clinical symptoms: *Bartonella bacilliformis*, *Bartonella quintana*, and *Bartonella henselae*.^5^, ^21^

Until 1993, *B. bacilliformis* was considered the only species of this genus. It is the etiologic agent of Carrion's disease, previously known as the only bartonellosis. *B. bacilliformis* is transmitted by the female *Lutzomyia verrucarum*, endemic in the Peruvian Andes and regions of Ecuador and Colombia.

Reports in recent decades of outbreaks in regions of atypical altitude strongly suggest epidemiological areas as potential for expansion. Current climate changes associated with human activities have contributed to the resurgence of infection and its expansion into new areas. Climate changes affect vector distribution and, additionally, phenomena such as El Niño have caused an increase in humidity levels, which favors the reproduction of vectors and the occurrence of outbreaks.^22^, ^23^ Some studies envolving animals to search for potential new hosts have shown that some species of apes in the jungles of South America, such as the Feline Night Monkey (*Aotus infulatus*), are susceptible to *B. bacilliformis* infection. These data warn of the risk of expansion of Carrion's disease due to the possible adaptation of vectors in areas inhabited by these animals, which may serve as disease dispersal facilitators in neighboring endemic regions, including Brazil.^24^

The disease is biphasic, with an acute phase (Oroya fever) characterized by fever, hemolytic anemia, and transient immunodeficiency and a chronic phase (Peruvian wart) marked by cutaneous vasoproliferative lesions.^1^, ^25^

The acute phase of the disease lasts from one to four weeks and severity can range from mild to fatal. Absence of antibiotic treatment can lead to a mortality rate of up to 88%. This is caused by the massive invasion of erythrocytes and initially leads to non-specific symptoms such as malaise, drowsiness, headache, chills, fever, anorexia and myalgia, which make the patient increasingly more jaundiced and confused. As the disease progresses, a severe hemolytic condition, accompanied by lymphadenopathy and hepatosplenomegaly, is established. Disease worsening can lead to acute respiratory distress, pericardial effusion, myocarditis, endocarditis, delirium, seizures, coma and multiple organ failure.^1^, ^9^, ^25^

After an average of two months in the acute febrile phase (which may not occur, particularly in natives of the endemic region) the Peruvian wart appears, an eruptive cutaneous manifestation formed by angiomatous lesions, which is often clinically and histologically similar to lesions of bacillary angiomatosis (BA). These lesions may present as angiomatous lesions, papules, papule-tumors, or nodules. They appear in patches, predominantly on the face and extremities, and measure 0.2–4 cm in diameter. They may persist for months or even years, and can be accompanied by fever, bone, and/or joint pains. The severity of the eruption is variable and it appears not to be related to previous antibiotic treatment. This is the tissue phase of Carrion's disease and is self-limiting.^26^ Although not fatal, if left untreated, these lesions persist as pathogen reservoirs and a source of contagion through the vector. This infection is usually treated with rifampicin, although streptomycin is also effective and was the drug of choice before 1975. Peruvian wart does not respond to treatment with chloramphenicol or penicillin. Treatment alternatives include ciprofloxacin and azithromycin associated with deflazacort.^27^ It does not lead to scarring, except when there is secondary infection.^28^, ^29^

Histologically, Peruvian wart lesions show a proliferation of endothelial cells of the terminal vasculature in the dermis and subcutis. The acute and chronic inflammatory infiltrate that accompanies the presence of *B. bacilliformis* in the interstice and inside the endothelial cells is an important finding, even in non-ulcerated lesions. The lesions can have more differentiated and ectatic vessels that are clinically and histologically similar to pyogenic granuloma. Cellular atypia can be seen, particularly in more solid lesions, with imperceptible lumens and spindle cells that resemble Kaposi sarcoma.^30^

*B. quintana* was initially associated with trench fever (TF), characterized by recurrent febrile episodes. Currently reported in hikers, alcoholics, and AIDS patients in the United States and Europe, the disease has been considered as re-emergent and is the agent implicated in cases of chronic bacteremia, endocarditis, and BA. Humans are the only known reservoirs and the transmission among them is through body lice, the reason why this pathogen is strongly associated to unsanitary conditions and poor personal hygiene.^31^ The disease is also known as quintana fever or five-day fever, and it has an incubation period of 15–25 days. TF can be asymptomatic or severe. Approximately half of those affected experience a sudden onset of flu-like symptoms with no respiratory symptoms and short duration. High and prolonged fever can occur over several weeks. Symptoms remit for many days and after an asymptomatic period there can be paroxysmal clinical exacerbation three to five times or more within a year.^30^ Eighty to 90% of patients present with erythematous, maculopapular lesions of up to 1 cm on the trunk.^32^ Furred tongue, conjunctival congestion, and musculoskeletal pain are frequently associated.^33^

**Figure 1:**
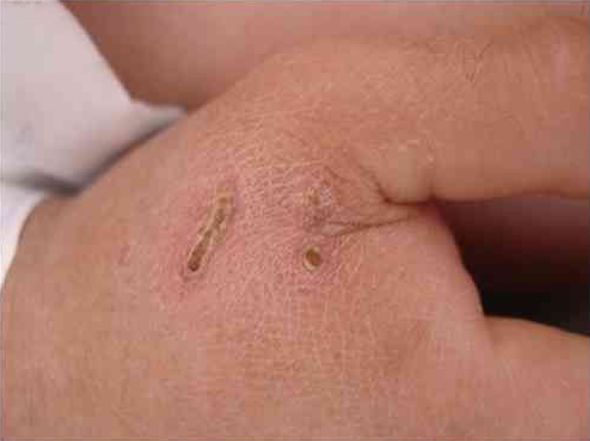
Lesions caused by cat scratches presented by a 28-year-old man with *Bartonella* sp. infection detected by polymerase chain reaction.

*B. henselae* is a zoonotic agent whose main reservoir is domestic cats. Transmission between cats does not occur in the absence of fleas, although transmission to humans is often associated with cat scratches. Fig. 1 shows the cat scratch observed in a 28-year-old man with Type I diabetes, presenting with nausea and vomiting for three days and lowering of consciousness for one day. He had Glasgow 3 level of consciousness and sepsis of unknown origin. There were two injuries that suggested scratching lesions. Infection by *Bartonella* sp. was detected through conventional polymerase chain reaction (PCR) for the internal transcribed spacer (ITS) region from a blood sample.

Contact with cats is a risk factor for *B. henselae* infection.^34^ Cats living in warm and humid geographical areas have a higher number of potential vectors and higher levels of bacteremia (7–%–43%) and anti-*B. henselae* seroprevalence (4%–81%).^25^, ^35^ This suggests that *Bartonella* sp. infection could be more prevalent in developing tropical countries. In Campinas, SP, Brazil, 90.2% of the cats involved in the study were positive for the test detecting the presence of *B. henselae* DNA in their blood.^36^

Besides cats, other pets already described as reservoirs include guinea pigs, rabbits, and dogs.^37^

Ticks have been proven to be vectors^37^ and contact with these arthropods has been associated with *Bartonella* sp. infection in blood donors from Hemocentro at Unicamp (Campinas, SP).^34^

Immunocompetent patients infected with *B. henselae* can develop cat scratch disease (CSD), characterized by a self-limited regional lymphadenitis associated with fever.

For Bass et al.^38^ in their review, CSD incidence is proportional to the density of the cat population, their ages, and human exposure to these animals. The authors also related the incidence of the disease to the prevalence and degree of infestation by fleas, *Ctenocephalides felis*, to warm and humid climates, related to geographical location and seasonality, reinforcing that the disease is more prevalent in tropical regions.

Lymphadenitis follows the primary lesion, from a few days to many weeks after the cat scratch or bite, apparently by the exposure of the dermis to bacteria found in the feces of feline fleas. It is characterized by an erythematous, non-pruritic papule on the area of the trauma or on its extremity, in case of a scratch. In 2–3 days it becomes vesicular and crusty, remaining for a few days and evolving to a patch that can last for up to 2–3 months. The lesion persists for 7–21 days or is sometimes present with lymph node enlargement. Rarely, the cutaneous lesion is the only clinical manifestation, even when there is history of a scratch or bite. The presence of the inoculation lesion should be thoroughly sought in the history and physical examination, since it can be found in over 90% of cases.^25^ After this period there can be superficial scarring similar to that of varicella. It may measure from a few millimeters to 1 cm in diameter.^30^

The histopathology of cutaneous lesions mimics that of lymph nodes, with the formation of granulomas with a central necrotic area, surrounded by lymphocytes and histiocytes and with a neutrophilic infiltrate. The pus can be loculed, which is important during aspiration. It differs from other granulomatous diseases with the presence of concurrent microabscesses and granulomas.^39^ Histopathological findings in lymph nodes can be mistaken for those seen in Hodgkin's disease, including cells similar to Reed–Stenberg cells.^40^ Microabscesses with bacterial clusters identified with the Warthin–Starry staining may be observed, mainly on newer lesions.^26^

Although rare, purpura can be serious.^41^ Maculopapular exanthem, erythema multiforme, and erythema nodosum are the cutaneous manifestations that, for Warwick,^42^ accompany CSD. For that author, erythema nodosum is the most frequent, to which Carithers,^43^ who does not see this association as a surprise, agrees, since erythema nodosum appears in the course of other granulomatous diseases such as tuberculosis and sarcoidosis. Erythema nodosum can occur in association with typical cases but usually appears associated with diffuse and non-regional lymph node enlargement.^44^

*B. henselae* also causes a wide variety of clinical conditions, such as fever of unknown origin, splenic and hepatic manifestations, encephalopathies, ocular diseases, endocarditis, *etc.*^1^

Patients infected by *B. quintana* or *B. henselae*, particularly those who are immunodeficient, can develop BA, which is characterized by angioproliferative lesions.^21^ Specifically in cases of *B. henselae* infection, these injuries may also be associated with peliosis, a rare condition characterized by small blood-filled cystic spaces found in the liver, often diagnosed only through biopsy, which may cause liver failure or rupture and may even be fatal.^21^, ^45^ Bacillary peliosis can also affect other organs.^46^

**Figure 2:**
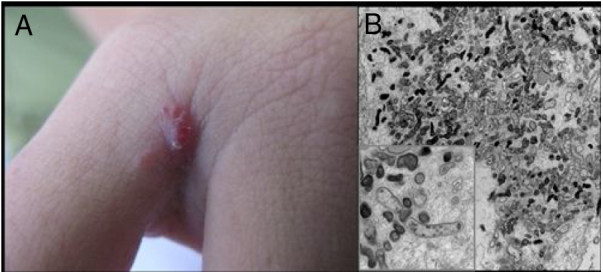
Bacillary angiomatosis: (A) single angiomatous lesion on the third interdigit of the right hand of a woman; (B) electron microscopy of cutaneous fragment transmission with innumerable Gram-negative bacilli featuring intra- and extracellular distribution (1200×, inset 16,000×).

Cutaneous lesions are the main manifestations of BA but the disease may not affect the skin in up to 45% of cases.^47^ They may be solitary lesions, but, more frequently, are multiple and widespread. They may be papules, plaques, angiomatous tumors, rarely hyperkeratotic, or nodules with skin-colored surface. A scaling collarette on the base of the lesion is a typical feature. They resemble pyogenic granuloma. They are friable and can bleed easily and profusely. The presentation of hardened and hyperpigmented plaques is the least frequent. There are reports of involvement in the oral, anal, conjunctival, gastrointestinal, and female genital mucous membranes, as well as airways. BA can be accompanied by disseminated visceral disease both in immunodeficient and immunocompetent individuals.^38^, ^48^, ^49^ Fig. 2 shows the case of a single angiomatous lesion in the third interdigit of the right hand of a 26-year-old woman presenting with fever, oral candidiasis, and weight loss for 2 months. Anti-HIV serology was reagent. Anatomopathological examination was compatible with bacillary angiomatosis and Warthin–Starry staining showed bacterial clumps. Gram-negative bacilli were observed through the analysis of a skin fragment using transmission electron microscopy.

Differential diagnosis with Kaposi sarcoma can be clinically impossible, particularly with early sarcomatous lesions. Both diseases can occur at the same time. Any other angiomatous lesions will be part of the differential diagnosis.^26^, ^30^

Regarding histology, there are three main features: (1) angioproliferation in lobules, with vessels formed by prominent endothelial cells, with atypia and mitoses being seen in areas with dense cellularity; (2) predominance of neutrophils in the inflammatory infiltrate and occasional leukocytoclasia; (3) presence of interstitial or intracellular bacterial clumps found with Warthin–Starry staining, immunohistochemistry, transmission electron microscopy, or confocal microscopy.^26^, ^30^, ^50^

It has been suggested that the difference between the angiogenic and granulomatous response triggered by the organism observed in BA and CSD, respectively, appears to be determined by the degree of the host's immunocompetence.^51^, ^52^ The concurrence of lesions with clinical and pathological features of CSD and BA, also reported after the use of corticosteroids, with the demonstration of the same agent, supports the above interpretation.^53^

**Figure 3:**
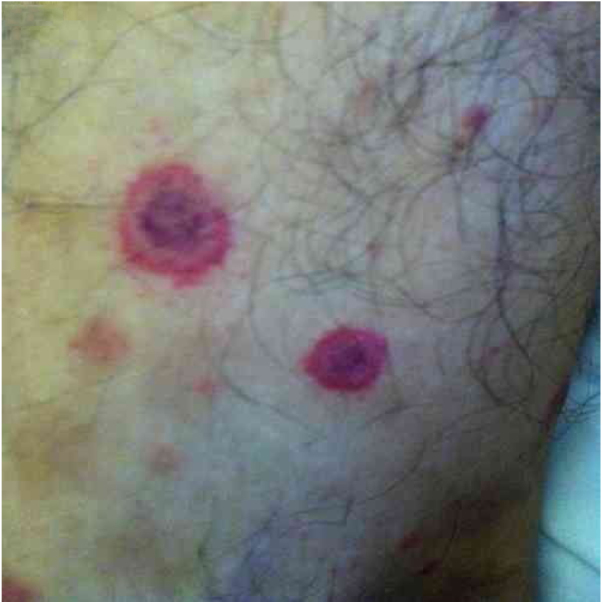
Cutaneous vasculitis on the leg of a 42-year-old man with a history of cat scratches and fever, with a diagnosis of *B. henselae* endocarditis confirmed by polymerase chain reaction, serology, and culture.

*Bartonella* spp. can cause asymptomatic cyclic bacteremia in humans and animals. This chronic infection can potentially result in endocarditis and be fatal.^1^ Nearly 31% of endocarditis cases have negative cultures and of those, up to 30% are caused by *Bartonella* spp.^20^ Six species of *Bartonella* have been associated with endocarditis, but 95% of endocarditis cases from these agents are caused by *B. quintana* or *B. henselae*.^54^ Vasculitis can occur and even simulate systemic vasculitis with antineutrophil cytoplasmic antibodies (ANCA) positivity (Fig. 3).^55^ Fig. 3 shows a case of skin vasculitis seen in a 42-year-old white male with a history of cat scratches and fever for 2 months. Skin lesions had appeared on his legs two weeks earlier. The diagnosis of endocarditis caused by *B. henselae* was confirmed by serology, PCR and culture.

*B. henselae* can cause chronic non-specific hepatic inflammation in adults and children. It can also be responsible for hepatic angiomatosis and bacillary peliosis, besides granulomatous hepatitis, with or without necrosis. *Bartonella* spp. are not included in guidelines for the screening of cryptogenic hepatitis and it is possible that part of the 40% of *de novo* hepatitis cases that occur after liver transplants are related to infection by these bacteria.^56^

**Figure 4:**
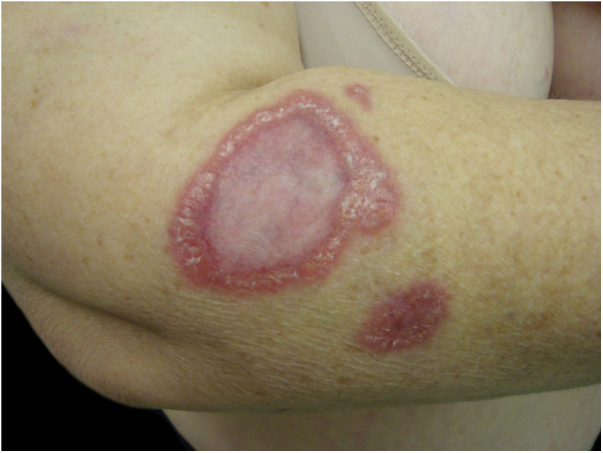
Annular granuloma presented by a 52-year-old woman. *B. henselae* DNA was amplified in a fragment of the mediastinal lymph node and in the patient's blood.

Often identified as the clinical expression of atypical CSD, non-classic forms of the disease should be considered separately, sch asmorbilliform exanthem, urticaria, erythema marginatum, granuloma annulare, leukocytoclastic vasculitis.^32^, ^41^ Fig. 4 shows a case of annular granuloma in a 52-year-old woman who reported a lesion similar to the image at the site of a cat scratch on her left forearm seven years earlier. The lesions spread. She had intense myalgia and arthralgia that made walking difficult. Chest and abdominal tomography showed mediastinal and retroperitoneal multiple lymph node enlargement. She had been treated with deflazacort 7.5 mg/d, methotrexate 15 mg/week and hydroxychloroquine 400 mg/d for 2 years, with a diagnosis of sarcoidosis. The anatomopathological examination of the skin was compatible with annular granuloma. *B. henselae* DNA was amplified in a fragment of a mediastinal lymph node and in the patient's blood.

**Figure 5:**
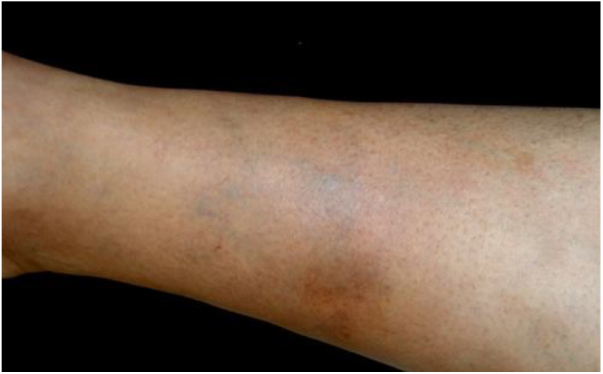
Sclerosing panniculitis with recurrent anemia. Sclerosing panniculitis in the right leg of a 32-year-old woman with a history of recurrent anemia of unknown origin. The patient subsequently tested for positive *B. henselae* DNA in blood samples.

Pyogranulomatous panniculitis was described in a dog whose owner had similar lesions. Both improved with treatment for *Bartonella* sp. infection.^57^ The authors followed a 32-year-old woman with sclerosing panniculitis, with a granulomatous reaction on her right leg detected during histological analysis and history of recurrent anemia of unknown origin, dependent on corticosteroids for 4 years. Electron microscopy showed Gram-negative bacteria inside an erythrocyte. Her case improved with erythromycin treatment. With the discontinuation of the antibiotic therapy after six weeks, the lesions recurred and no longer responded to antibiotic therapy. A blood sample from the patient was subsequently screened for *B. henselae* DNA, which showed to be positive (Fig. 5).

Cutaneous manifestations can appear whether or not associated with granulomatous manifestation on the liver, spleen, heart, bones, and mesenteric and/or mediastinal lymph nodes.^30^

A growing number of possible immune parainfectious or post-infectious manifestations have been described in association with *Bartonella* spp. infection.^41^ Considering the broad spectrum of the infection and the potential complications associated to *Bartonella* spp., the infection should be considered by physicians more frequently among the differential diagnoses of idiopathic conditions. The conditions that should include *Bartonella* sp. infection in the differential diagnosis are listed in Table 1.

**Table 1:** Idiopathic manifestations potentially associated to *Bartonella* spp. infection.

Prolonged fever
Recurrent or severe anemia
Hepatitis
Serositis
Chronic lymphadenopathy
Chronic fatigue
Uveitis
Retinitis
Neuritis
Febrile maculopapular exanthem
Purpura
Urticaria
Erythema nodosum
Erythema multiforme
Erythema marginatum
Granuloma annulare
Leukocytoclastic vasculitis
Granulomatous reactions
Angioproliferative reactions

### Possibility of transmission by blood transfusion

Since *Bartonella* spp. can cause asymptomatic infections, the extent of the infection might be underestimated. The worldwide seroprevalence of *Bartonella* sp. in humans ranges from 1.5% to 77.5%.^58^ In a study with 437 healthy patients from a rural region in Piau, MG Brazil, the seroprevalence was 12.8% for *B. quintana* and 13.7% for *B. henselae*.^59^ In another study conducted with 125 blood donors in Rio de Janeiro, 43 (34.4%) were seropositive for *B. henselae*.^13^

Asymptomatic hosts with erythrocytic infection can donate blood. In a recent study with 500 blood donors in Campinas, SP, Brazil, antibodies to *B. quintana* and *B. henselae* were detected in 32.0% (136/500) and 16.2% (78/500) of the donors, respectively. The same study found 3.2% of blood donors with *Bartonella* spp. blood infection; in 1.2% of them, *B. henselae* bacteremia was documented inthe donated blood.^3^

Blood transfusion represents a potential risk for the transmission of these bacteria. Cats were experimentally infected with *B. henselae* and *B. clarridgeiae* through intravenous and intramuscular inoculation with the blood of cats known to be infected.^60^ In addition, transmission through transfusion has been documented in immunocompetent mice.^7^ A study using transmission electron microscopy and culture documented the ability of *B. henselae* to survive in blood stored at 4 °C for 35 days.^61^ There are two reports of the transmission of the infection to humans through accidental percutaneous injection with contaminated blood.^62^, ^63^ The actual worldwide prevalence among blood donors is unknown and routine screening of donated blood is not conducted for these pathogens.

### Laboratory diagnosis

There is no standard laboratory diagnosis for infections caused by *Bartonella* spp. It is increasingly clear that none of the diagnostic methods available currently will confirm *Bartonella* sp. infection in all infected immunocompetent patients, since this group has low bacteremia, which makes detection even more difficult.^19^ This difficulty in laboratory diagnosis is another contributing factor for neglecting this pathogen. Nowadays, it is clear that multiple techniques must be used in combination to avoid false-negative results.^3^, ^64^ The most common laboratory diagnostic tools are indirect immunofluorescence (IIF) serology, culture, or PCR.^65^, ^66^, ^67^

IIF is the most common method because of its simplicity. However, immunologic methods have some limitations such as cross-reaction among species and with multiple pathogens, which can lead to false-positive results. There is also the possibility of false-negative results since the antigens from commercial kits are limited to a few species.^68^, ^69^ Other factors that should be taken into consideration are the heterogeneity among the strains and genotypes of *Bartonella* spp., the differences in analysis parameters among pathologists, and the subjectivity of reading the results with IFA. Many studies have demonstrated the lack of correlation between PCR and positive serology.^64^ In general, the serologic test should not be used as the only diagnostic tools and, in case of positivity, it should only be interpreted as past exposure to *Bartonella* sp. Serologic testing should be used with other techniques such as culture and PCR to assure diagnostic accuracy.^70^

The use of conventional microbiologic techniques to detect and isolate *Bartonella* spp. is not as efficient due to the fastidious nature of these bacteria, the low number of circulating bacteria in infected organisms, and the cyclical bacteremia. Isolation requires a long incubation period (six to eight weeks) and special growth conditions (special culture media enriched with blood above 35 °C, in a saturated water atmosphere with 5% CO_2_).^35^, ^71^, ^72^ Primary isolation is rarely successful in non-reservoir and/or immunocompetent hosts, as well as humans with CSD.^71^, ^72^, ^73^, ^74^ Liquid culture of *Bartonella* spp. increases detection sensitivity of infection by these bacteria *via* molecular methods.^72^, ^73^, ^75^

There is no consensus about the best primers and conditions to be used for detection of *Bartonella* DNA through PCR. Besides the primers that determine the region to be amplified and, therefore, the sensitivity of the reaction, the chosen PCR technique also influences the success of the diagnosis. Double amplification PCR can enhance detection sensitivity considerably, as well as real-time PCR.^35^, ^67^, ^75^, ^76^ The advantages of diagnostic molecular techniques such as PCR are fast results and the possibility of identification of the species causing the infection.^77^ Nonetheless, there are limitations, such as the possibility of false-positive results (through contamination of previously positive samples) or false-negative results (due to an amount of DNA inferior to the detection threshold). In addition, finding the pathogenic DNA in a sample does not necessarily guarantee an active infection.^78^, ^79^

Histology is not frequently used as a diagnostic method but can be very valuable for BA cases, Peruvian wart, and CSD, or when there is tissue involvement, even if not cutaneous. Cutaneous histologic findings were described above.

### Therapeutics

There is no therapeutic regimen that guarantees eradication of *Bartonella* from the organism. This can be easily demonstrated by the appearance of Peruvian warts even in patients treated with antibiotics for Oroya fever. Futhermore, antibiotic treatment does not alter the cure rates in patients with lymph node enlargement caused by *Bartonella* spp.^80^

Since there are no systematic reviews on this topic, treatment decisions are based in case reports that test a limited number of patients. Patients with systemic disease caused by *Bartonella* spp. should be treated with gentamicin and doxycycline;chloramphenicol has been proposed for treatment in case of bacteremia by *B. bacilliformis* (Carrion's disease). Gentamicin associated with doxycycline is considered the best treatment for endocarditis and TF, and rifampicin or streptomycin can also be used to treat Peruvian warts.^5^ Erythromycin is the antibiotic of choice for BA and hepatic peliosis cases; it should be administered for a minimum of two months.^1^

### Prevention

As mentioned previously, contact with cats is the main risk factor for transmission of CSD and other forms of bartonellosis. Flea infestations, free street access, and an environment with multiple cats are factors that increase the likelihood of feline infection. Therefore, cat owners should avoid flea infestation, keeping them indoors and away from stray cats. The European Advisory Board on Cat Diseases suggests that immunodeficient people adopt cats older than 1 year of age, with no fleas, in good general health, and that do not come from shelters or houses with multiple cats.^81^ To prevent TF, people should avoid contact with body lice and improve personal hygiene. Carrion's disease can be prevented by the use of repellents and clothing that protect from sand fly bites in areas where the disease is endemic.^82^

Besides these relevant preventive measures, dissemination of information on *Bartonella* sp. infection to the medical community in general is necessary to avoid the occurrence of bartonellosis. Neglecting the disease certainly contributes to the dissemination of the infection and to inadequately treated cases all over the world.

## Conclusion

Bartonellosis are associated with a broad spectrum of symptoms, debilitating conditions, and potentially fatal outcomes. Ectoparasites are involved in the transmission of *Bartonella* sp. These diseases are frequently neglected by health care professionals and researchers. The infection can be asymptomatic and have a great impact on the morbidity of, for example, patients with Hansen's disease (as triggers for Type 2 leprosy reaction), or patients with sickle cell anemia (associated with painful crisis due to vaso-occlusion), and cryptogenic hepatitis or cirrhosis. Diagnosis is challenging because physicians do not consider the possibility of bartonellosis and, even when this occurs, there are technical and laboratory difficulties for a conclusive diagnosis. There should be incentives for more research related to *Bartonella* spp. infection. There are limited resources for the investigation of these agents since Bartonellosis are not even in the list of neglected diseases of the World Health Organization.^83^ However, these diseases are amenable to being controlled, prevented, and even eradicated with plausible and effective measures.

## Financial support

Doctoral Scholarship from CNPq 159717/2013-2 (Drummond, MR); Productivity Grant from CNPq 301900/2015-9 (Velho, PENF).

## Author's contribution

Karina de Almeida Lins: Approval of final version of the manuscript; conceptualization and planning of the study; composition of the manuscript; critical review of the literature; critical review of the manuscript.

Marina Rovani Drummond: Approval of final version of the manuscript; composition of the manuscript; critical review of the literature; critical review of the manuscript.

Paulo Eduardo Neves Ferreira Velho: Approval of final version of the manuscript; conceptualization and planning of the study; composition of the manuscript; critical review of the literature; critical review of the manuscript.

## Conflicts of interest

None declared.
